# Contactless, nondestructive determination of dopant profiles of localized boron-diffused regions in silicon wafers at room temperature

**DOI:** 10.1038/s41598-019-46986-z

**Published:** 2019-07-18

**Authors:** Hieu T. Nguyen, Zhuofeng Li, Young-Joon Han, Rabin Basnet, Mike Tebyetekerwa, Thien N. Truong, Huiting Wu, Di Yan, Daniel Macdonald

**Affiliations:** Research School of Electrical, Energy and Materials Engineering, The Australian National University, Canberra, ACT 2601 Australia

**Keywords:** Solar cells, Electrical and electronic engineering, Fluorescence spectroscopy

## Abstract

We develop a photoluminescence-based technique to determine dopant profiles of localized boron-diffused regions in silicon wafers and solar cell precursors employing two excitation wavelengths. The technique utilizes a strong dependence of room-temperature photoluminescence spectra on dopant profiles of diffused layers, courtesy of bandgap narrowing effects in heavily-doped silicon, and different penetration depths of the two excitation wavelengths in silicon. It is fast, contactless, and nondestructive. The measurements are performed at room temperature with micron-scale spatial resolution. We apply the technique to reconstruct dopant profiles of a large-area (1 cm × 1 cm) boron-diffused sample and heavily-doped regions (30 μm in diameter) of passivated-emitter rear localized-diffused solar cell precursors. The reconstructed profiles are confirmed with the well-established electrochemical capacitance voltage technique. The developed technique could be useful for determining boron dopant profiles in small doped features employed in both photovoltaic and microelectronic applications.

## Introduction

An attractive approach for improving light-to-electricity power conversion efficiencies of crystalline silicon (c-Si) solar cells is to minimize surface areas of heavily-doped layers. This is due to the high recombination-active nature of the heavily-doped layers, causing a significant loss of photo-induced electrons and holes. Several solar cell designs employing this concept have been proved to achieve efficiencies over 24% such as interdigitated back-contact (IBC)^1–3^ and passivated-emitter rear localized-diffused (PERL)^4–6^ cell structures. However, once the heavily-doped regions are shrunk down to micrometer sizes, determining their dopant profiles is challenging. Common techniques to measure the dopant profiles - such as electrochemical capacitance voltage (ECV) and secondary-ion mass spectrometry (SIMS) - all have limitations due to either their spatial resolutions or destructive and time-consuming natures.

Photoluminescence (PL) spectra emitted from c-Si wafers and solar cells are complex functions of sample temperatures, surface morphologies, excess carrier profiles, and dopant densities, to name a few. Variations in any of these parameters can all affect their spectral intensities and shapes. However, from a different perspective, these spectral sensitivities open a unique opportunity to investigate important properties of c-Si wafers and solar cells by interpreting their PL spectra. Many applications of spectrally-resolved PL have been demonstrated for c-Si photovoltaics including the absorption coefficient and radiative recombination coefficient^7–10^ and bandgap^11,12^ of c-Si, diffusion lengths of minority carriers^13–15^ in c-Si wafers and bricks, or light-trapping capabilities of various plasmonic structures^16^. In addition, once equipped with confocal optics, namely micro-photoluminescence spectroscopy (µ-PLS), this class of techniques can give access to various microstructures such as grain-boundaries^17,18^, dislocations^19–21^, and metal/oxygen precipitates^22–25^.

Many authors have also utilized the µ-PLS-based techniques to study dopant densities of localized heavily-doped regions in c-Si wafers and solar cells. At room temperature, Gundel *et al*.^26^ and Woehl *et al*.^27^ measured PL peak energy shifts, caused by bandgap narrowing effects in heavily-doped c-Si, across vertical cross-sections of laser-doped regions and extracted their dopant densities (>1 × 10^18^ cm^−3^). This approach was destructive and required very thick junction depths (e.g. aluminum-alloyed regions) as the excitation spot size was ~1 micron in diameter and they had to scan across the vertical cross-sectional areas. Heinz *et al*.^28^ and Roigé *et al*.^29^ employed a different approach – capturing PL spectra from above sample surfaces and observing changes in PL intensities or spectral shapes, respectively, from heavily-doped regions (>1 × 10^18^ cm^−3^). Doing this, they could avoid damaging their samples. However, as this approach used heavily-doped c-Si wafers (uniform across the wafer thickness) for calibrations, it was challenging to extract entire dopant profiles of thermally-diffused layers whose dopant densities varied across their thicknesses. Also, using the entire spectrum, the technique, in principle, could be affected by photon reabsorption.

At low temperatures (~80 K), Nguyen *et al*.^30^ reported a distinct PL peak emitted from typical thin diffused regions for solar cells, in addition to the common PL peak emitted from c-Si substrates. Later, Han *et al*.^31^ found that the energy and intensity of this new peak (relative to the c-Si substrate peak) were correlated with the peak dopant density and thickness of the boron-diffused layers, respectively. Employing a set of boron-diffused samples for calibrations, the authors extracted dopant profiles of various localized p+ regions. However, performing measurements at 80 K limits the use of this technique as a fast characterization method. To overcome this limitation, Nguyen *et al*.^32^ carried out measurements at room conditions. However, due to thermal broadening effects, the two PL peaks from the substrate and the p+ layer (observed at 80 K) were not distinguishable anymore. Therefore, the authors could not extract the dopant profiles, but rather sheet resistances of localized p+ regions.

In this paper, employing two different excitation wavelengths, we further advance the µ-PLS-based techniques reported by Han *et al*.^31^ (dopant profiles, 80 K) and Nguyen *et al*.^32^ (sheet resistances, room temperature) to reconstruct entire dopant profiles of localized boron-diffused regions on c-Si wafers and solar cell precursors at room temperature. First, we describe the experimental details and explain the underlying principle of our method. We then examine correlations between various parameters of dopant profiles and room-temperature PL spectra from boron-diffused samples. After that, we establish calibration curves based on these correlations and use them to reconstruct dopant profiles of a standard boron-diffused sample and localized p+ regions of a PERL cell precursor. Finally, we discuss some practical limitations of the technique.

## Experimental Details

Fifteen boron-diffused samples fabricated from float-zone (FZ) 100-Ω.cm n-type Si wafers were used to establish calibration curves. The diffusion was achieved via two steps – a quartz tube-furnace deposition step followed by wet-chemical etching to remove the boron-rich layer, and a subsequent high-temperature drive-in step. Their sheet resistances were from 20 to 200 Ω/ϒ, covering a practical range for photovoltaic applications. A PERL solar cell precursor was fabricated from a FZ 5-Ω.cm p-type Si wafer. A dielectric diffusion mask made of a stack of a thermally-grown SiO_2_ layer (∼30 nm) and a low-pressure chemical-vapor deposition (LPCVD) Si_3_N_4_ layer (∼80 nm) was formed on both sides of the wafer. Localized openings (30-μm diameter) were created on one side using a photolithography process. It then went through the two-step diffusion process to form localized-doped regions. The resultant dopant profile was measured via an ECV profiling technique on additional 1 cm × 1 cm dielectric openings on the patterned wafer. The dielectric mask and residual borosilicate glass layer were subsequently removed by a concentrated HF solution prior to PL measurements.

The employed µ-PL/Raman system was a Horiba LabRAM system equipped with confocal optics and an X-Y micro-mapping stage. PL spectra were captured with a liquid-nitrogen-cooled InGaAs array detector (detection range of 750–1600 nm) and a supercontinuum NKT laser source (tunable wavelength range of 480–2000 nm). Two excitation wavelengths (500 and 600 nm) were used in this work. Raman experiments were conducted with a charge-coupled-device (CCD) Si array detector (detection range of 400–1000 nm) and a single wavelength solid-state 532-nm laser. The laser light was focused onto the sample surface via a 50× objective lens (numerical aperture = 0.55). The on-sample illumination spot size was ~1 µm and the on-sample power was kept constant at 1.5 mW. The spectral response of the entire system was determined with a calibrated halogen-tungsten light source.

## Results and Discussions

**Figure 1:**
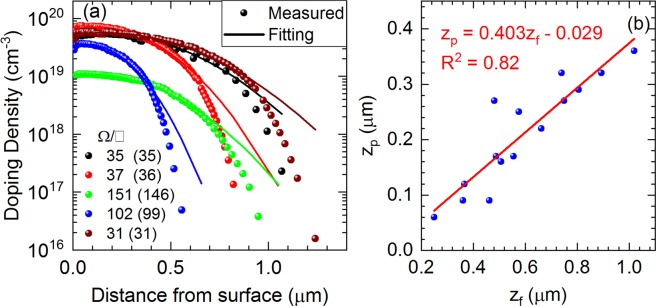
(**a**) ECV dopant profiles of some calibration samples and their corresponding Gaussian fits using Eq. 1. The sheet resistances calculated from these measured and fitting profiles are also shown (outside and inside the bracket, respectively). (**b**) Correlation between *z*_*f*_ and *z*_*p*_.

First, we describe the principle of employing PL spectra to determine dopant profiles of boron-diffused regions. Figure 1a shows dopant profiles, measured by the ECV technique, of a selected set of boron-diffused calibration samples. As the boron-rich layer was removed before the final thermal drive-in process (i.e. the dopant source is finite), each dopant profile can be fitted with a Gaussian function^33^:

1 $$N(z)={N}_{p}\times exp[\frac{-{(z-{z}_{p})}^{2}}{{{z}_{f}}^{2}}]$$

where *N*_*p*_ is the peak dopant concentration, *z*_*p*_ is the depth at which the peak locates, and *z*_*f*_ is a depth factor. Deviations between the measurements and fittings (see Fig. 1a) correspond to the Gaussian tails. They happen at low dopant densities (one order of magnitude lower than the peak concentration) and do not affect the fitting quality significantly. The sheet resistances calculated from these measured and fitting profiles are shown to match (outside and inside the bracket, respectively). In addition, *z*_*f*_ and *z*_*p*_ inherently correlate due to their similar natures in the two-step diffusion process – the deeper the peak concentration is, the more the dopant atoms diffuse into the substrate – as shown in Fig. 1b. Therefore, it is possible to reconstruct the dopant profiles using Eq. 1 given that PL spectra from boron-diffused regions can be converted into the parameters {*N*_*p*_, *z*_*f*_, *z*_*p*_}.

**Figure 2:**
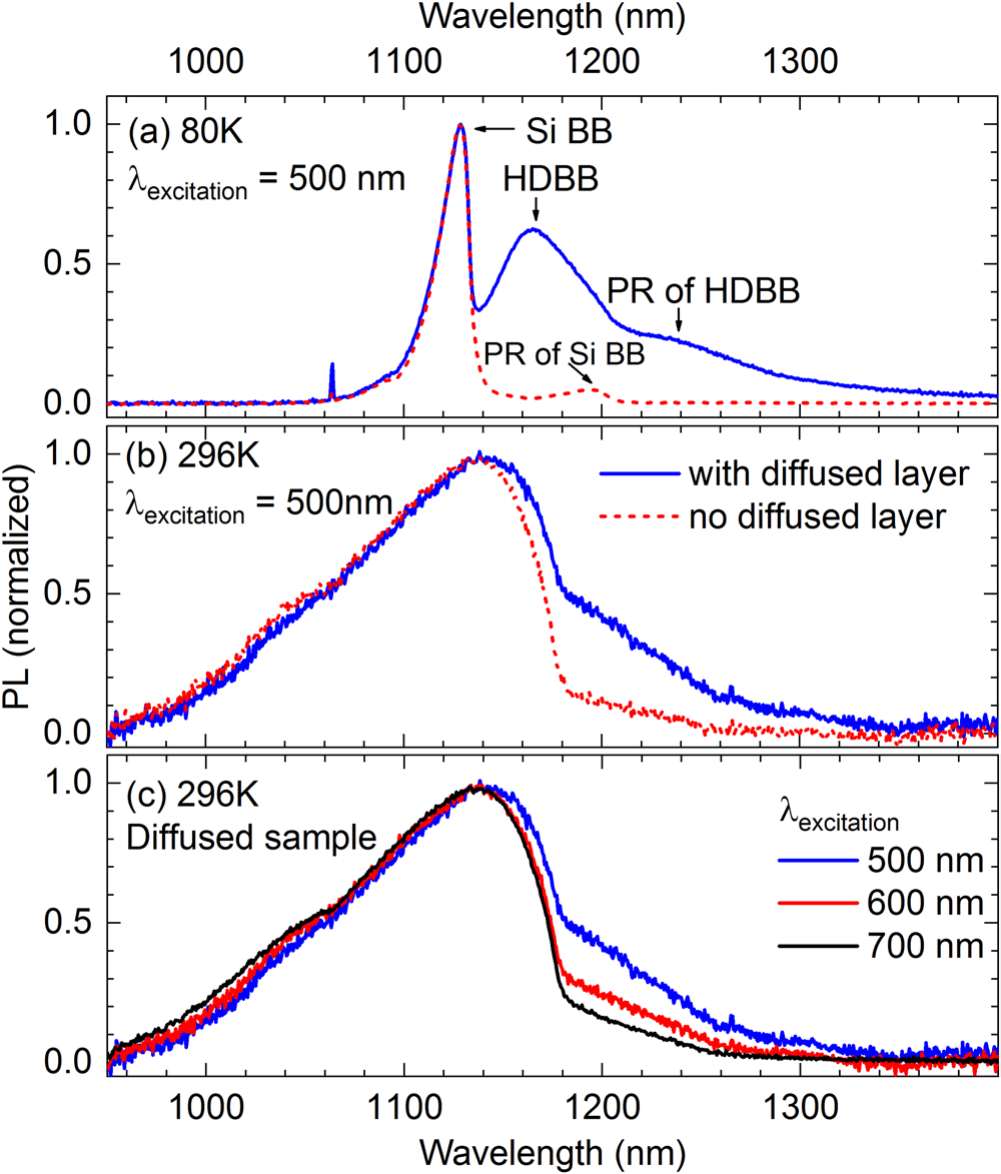
Comparison of normalized PL spectra from c-Si samples with and without a diffused layer at (**a**) 80 K and (**b**) 296 K, captured with the 500-nm excitation wavelength. (**c**) PL spectra from the diffused wafer with various excitation wavelengths at 296 K.

As shown in Fig. 2a, a lightly-doped c-Si substrate exhibits two main PL peaks at 80 K – the one located ~1130 nm is the main band-to-band emission (Si BB) and the other located ~1200 nm is its zone-center optical photon replica (PR of Si BB)^34^. For a diffused wafer, the 500-nm excitation light is absorbed in both its diffused layer and c-Si substrate, thus the emitted spectrum contains signatures of both layers. Compared to the c-Si substrate, the diffused layer yields two peaks ~1160 nm and ~1230 nm associated with its main band-to-band emission and phonon replica, respectively. These two peaks have lower energies (i.e. longer wavelengths) than those of the substrate since the bandgap is narrowed in heavily-doped Si^12^. We denote these two peaks as “HDBB” (heavily-doped band-to-band) and “PR of HDBB” in Fig. 2a. The peak wavelength location and relative intensity of the HDBB peak strongly depend on the dopant profiles of the diffused layers. Han *et al*.^31^ utilized these two spectral properties to extract the parameters *N*_*p*_ and *z*_*f*_ and then reestablished the dopant profiles by capturing PL spectra at 80 K with a single excitation wavelength.

At room temperature, although the effect of the diffused layer is still noticeable at the high-energy (long-wavelength) spectral region, the two main band-to-band PL peaks (one from the diffused layer and one from the c-Si substrate) are indistinguishable due to thermal broadening effects (Fig. 2b). In this case, an integrated intensity ratio between 1135–1250 nm (dominated by the diffused layer) and 1115–1135 nm (the spectral peak region) can be used as a metric for assessing the diffused layer. This ratio was demonstrated to be unaffected by photon reabsorption and thus by the wafer surface geometry and thickness^32^. Therefore, the measurements can be done on both planar and textured surfaces. Moreover, by varying excitation wavelengths, this intensity ratio varies significantly due to the different penetration depths of the light in the sample (Fig. 2c). The longer wavelength light can penetrate more deeply into the substrate, leading to a lower impact of the diffused layer on the overall spectrum and thus reducing the intensity ratio. This relative change in intensity ratio versus excitation wavelength can be used as another spectral parameter to extract {*N*_*p*_, *z*_*f*_, *z*_*p*_}.

**Figure 3:**
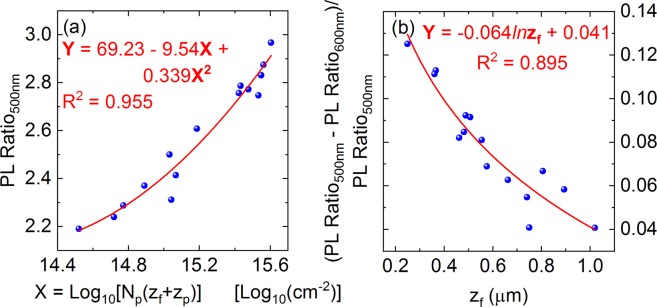
(**a**) PL intensity ratio versus *N*_*p*_ × *(z*_*f*_ + *z*_*p*_) excited with the 500-nm light. (**b**) Relative change in PL intensity ratio between 500-nm and 600-nm excitation light versus *z*_*f*_. The PL ratio is taken between the integrated intensities of 1135–1250 nm and 1115–1135 nm. T = 296 K.

Next, we assess relationships between the integrated PL intensity ratio defined above and the parameters {*N*_*p*_, *z*_*f*_, *z*_*p*_} for all calibration samples. Figure 3a shows the PL ratios captured with the 500-nm excitation light versus the product of *N*_*p*_*×(z*_*f*_ + *z*_*p*_). Figure 3b shows the relative change of the PL ratios captured between 500-nm and 600-nm excitation light versus the depth factor *z*_*f*_. Here, the quantity *N*_*p*_ × *(z*_*f*_ + *z*_*p*_) was chosen due to the fact that PL intensities directly relate to the dopant density and the layer thickness. Multiple attempts were performed to find correlations between various combinations of PL ratios and {*N*_*p*_, *z*_*f*_, *z*_*p*_} (Table 1) and the two pairs in Fig. 3a,b were found to have the strongest correlations. We can fit the correlations observed in these figures with two calibration curves. The two curves, along with the calibration curve in Fig. 1b, can be employed to determine {*N*_*p*_, *z*_*f*_, *z*_*p*_} of a certain boron-diffused Si sample by capturing its room-temperature PL spectra with the 500-nm and 600-nm excitation light. Then, one can use Eq. 1 to reestablish the dopant profile of this sample. Note that the parameterization curves (fitting functions) chosen in Fig. 3 and Table 1 are only arbitrary choices to fit the experimental data. One can employ various functions for these fitting curves. In our study, we found that the 2^nd^-order polynomial and logarithmic functions gave good fits.

**Table 1:** Correlation factors (R^2^) between PL intensity ratios and various combinations of {N_p_, z_f_, z_p_}.

Parameters	PL ratio λ_exc_ = 500nm A	PL ratio λ_exc_ = 600 nm B	Relative change in PL ratio (A-B)/A	Change in PL ratio A-B	Fitting function
log(N_p_ × (z_p_ + z_f_))	0.96	0.85			aX^2^ + bX + c
log(N_p_ × z_p_)	0.92	0.92			aX^2^ + bX + c
log(N_p_ × z_f_)	0.93	0.82			aX^2^ + bX + c
log(N_p_)	0.59	0.4			aX^2^ + bX + c
z_p_			0.75	0.6	−a*ln*(X) + b
z_f_			0.9	0.77	−a*ln*(X) + b
z_p_ + z_f_			0.88	0.75	−a*ln*(X) + b

The R^2^ values are obtained from fitting the measured data with a polynomial function Y = aX^2^ + bX + c or a logarithmic function Y = −a*ln*(X) + b. The PL intensity ratios are taken between the spectral integrated intensities of 1135–1250 nm and 1115–1135 nm. The blank cells are where the pairs do not display any correlation. T = 296 K. λ_excitation_ = 500 and 600 nm.

**Figure 4:**
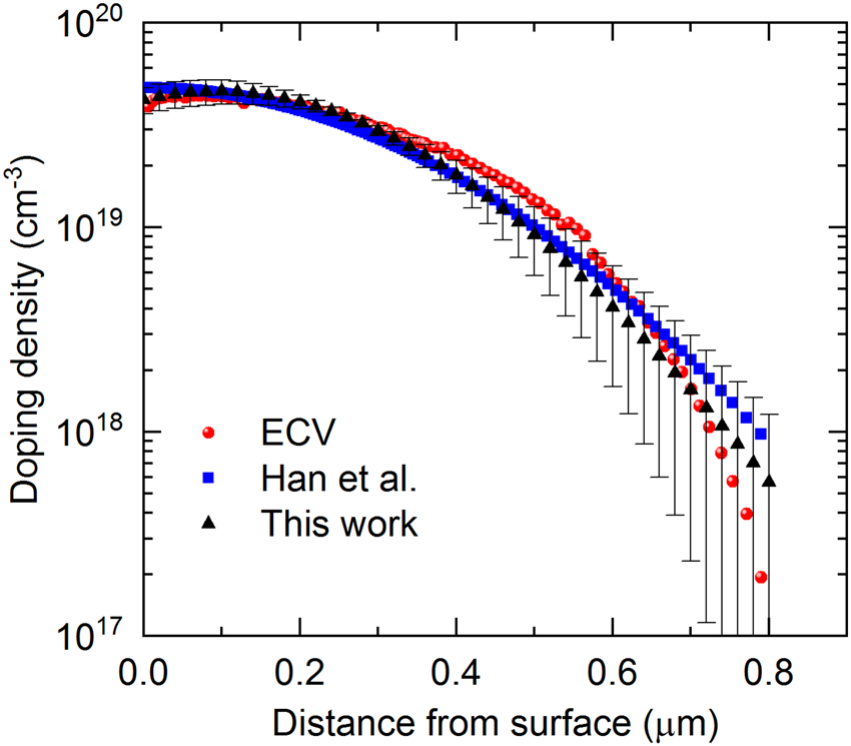
Comparison of dopant profiles among ECV measurements (red circles), Han *et al*.’s method at 80 K^31^ (blue squares), and this work at room temperature (black triangles). The error bar is ± one standard deviation, obtained from fifteen data points in the 1 cm × 1 cm diffused area of the patterned wafer.

Now, we demonstrate that the technique can be used to reestablish dopant profiles of boron-diffused layers. We capture room-temperature PL spectra from various locations in the 1 cm × 1 cm diffused area on the patterned wafer used for the PERL solar cell precursor. Two spectra (with 500-nm and 600-nm excitation wavelengths) are used to extract a dopant profile at each location. Profiles obtained from fifteen locations are averaged to yield the final profile, along with a standard deviation at each depth location. We also apply the technique reported by Han *et al*.^31^ at 80 K (single excitation wavelength at 532 nm) to determine the dopant profile of this 1 cm × 1 cm diffused area. The actual profile is then measured using the ECV method. Figure 4 compares the results among the three techniques. Both the μ-PLS-based techniques yield very similar results to the ECV one. From the figure, larger errors happen at lower doping densities. This is due to the lower weighting of the low dopant tail in the Gaussian fits in Fig. 1.

**Figure 5:**
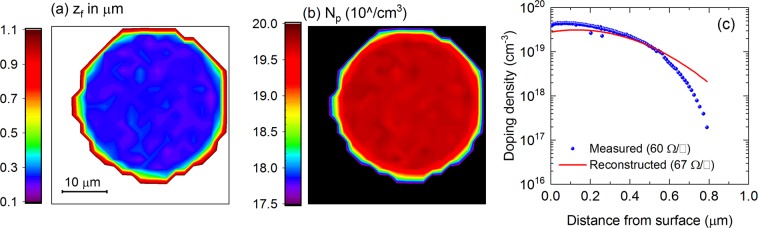
(**a**) z_*f*_ and (**b**) *N*_*p*_ maps of a localized p+ region in the PERL solar cell precursor, determined by the µ-PLS method at room temperature. (**c**) Comparison between the ECV-measured and reconstructed dopant profiles. The measured profile was done on the 1 cm × 1 cm p+ test region on the same sample. The calculated sheet resistances are shown in the bracket.

Next, we apply the technique to determine dopant profiles in localized p+ regions of the PERL solar cell precursor. We perform μ-PLS mapping around a localized p+ region using the 500-nm and 600-nm excitation wavelengths. The μ-PLS map provides an entire spectrum for every pixel in the X-Y map, allowing an extraction of the PL intensity ratio with micron-scale spatial resolution. Based on two PL intensity ratios (with 500-nm and 600-nm excitation light) of every single pixel, we can extract *N*_*p*_, *z*_*f*_, and *z*_*p*_ maps determined from the calibration curves in Figs 3 and 1b. The results are shown in Fig. 5a,b for *z*_*f*_ and *N*_*p*_, respectively. The reconstructed dopant profile at the center of the p+ region reasonably matches the measured dopant profile on the test location (1 cm × 1 cm diffused region). The sheet resistances calculated from the reconstructed and measured dopant profiles are 67 and 60 Ω/ϒ, respectively. These results demonstrate that this µ-PLS-based method can be applied to extract dopant profiles of localized boron-diffused regions with very high spatial resolution.

**Figure 6:**
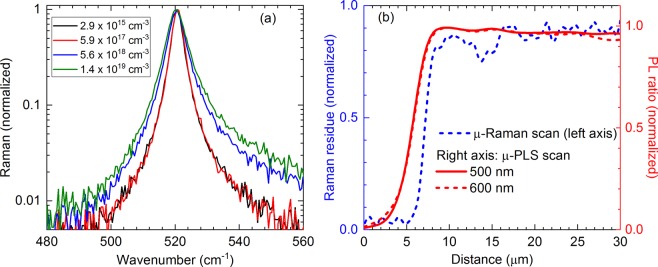
(**a**) Raman spectra of c-Si wafers with various uniform dopant levels depth-wise. Higher dopant levels yield broader c-Si Raman peaks. (**b**) Comparison of line scan profiles across the p+ circle in Fig. 4b between the μ-Raman and μ-PLS measurements. The Raman residue is the integrated intensity difference between the normalized Raman peaks from the p+ circle and the normally-doped region. T = 296 K.

However, we observe an inhomogeneity of dopant profiles around the edge of the p+ circle. We continue performing μ-Raman scans to verify whether this non-uniformity is caused by real dopant variations or by carrier smearing effects in the µ-PLS experiments. In Raman measurements, there is no carrier smearing as excited electrons are still bound to their host atoms or molecules, thus increasing the lateral spatial resolution. Moreover, a high dopant level of boron has been known to broaden the Raman spectrum of c-Si due to Fano resonance effects^29,35–38^. Higher dopant densities yield broader c-Si Raman peaks (Fig. 6). Therefore, we utilize this phenomenon to verify the apparent non-uniformity observed in the µ-PLS results. The μ-Raman scanning profile across the p+ circle has a much sharper edge than the µ-PLS profile (Fig. 6). These results show that, for the small-area p+ regions investigated, our μ-PLS system’s spatial resolution has a limitation of ~3 µm near their edges, a fact that has been already confirmed by our previous work on mapping sheet resistances using the μ-PLS approach^32^.

Finally, we note that the degree of impacts of the diffused layers on PL spectra varies with various experimental setups due to different generation (in the samples) and detection (of the system) profiles. For examples, variations in excitation wavelengths will change the fraction of the laser light absorbed in the diffused layer and the substrate; the system will be more surface sensitive with a higher numerical aperture objective lens and a smaller detection pinhole; various laser intensities can also yield different PL spectra due to different injection level dependences of PL signals from the diffused layer and the substrate. Therefore, the established calibration curves in Fig. 3 are only valid for the specific experimental configuration employed in this study. These limitations are valid for not only this work, but also numerous works applying μ-PLS methods on heavily-doped layers in c-Si wafers^17,18,26–32^. In addition, as our method relies on typical Gaussian shapes of dopant profiles in standard boron-diffused layers, the calibration curves cannot be applied on heavily-doped layers prepared by other techniques such as laser doping, ion implantation, or epitaxial growth. Dopant profiles from these techniques do not follow Gaussian distributions.

## Conclusion

In summary, we have developed a photoluminescence-based method to quickly reconstruct dopant profiles of localized boron-diffused regions in crystalline silicon solar cells using two excitation wavelengths, 500 and 600 nm. This newly-developed technique employs the sensitivity of photoluminescence spectra on dopant profiles of boron-diffused regions, courtesy of bandgap narrowing effects in heavily-doped silicon. In addition, it utilizes various penetration depths of the two excitation wavelengths in silicon. Although our new technique requires a set of calibration samples the same as other established micron-scale techniques, it has several advantages. It is contactless and nondestructive. The measurements can be readily performed at room temperature, on typical diffused layers in solar cell applications (thin and non-uniformly doped), and with micron-scale spatial resolution. Finally, we have also successfully demonstrated the technique on a PERL solar cell precursor.
